# Predictive parameters of semen outcome improvement after varicocele treatment by scleroembolization: a retrospective study

**DOI:** 10.1007/s40618-026-02844-0

**Published:** 2026-04-29

**Authors:** Luca De Toni, Giordana Ferraioli, Matteo Todisco, Sara Congiu, Pierfrancesco Palego, Andrea Graziani, Nicola Caretta, Giuseppe Grande, Giorgio De Conti, Massimo Lafrate, Fabrizio Dal Moro, Alberto Ferlin, Andrea Garolla

**Affiliations:** 1 Department of Medicine, University of Padova, Padova, Italy; 2 Unit of Radiology, Padova University-Hospital, Padova, Italy; 3 Unit of Andrology and Reproductive Medicine,Department of Systems Medicine, University Hospital of Padova, Padova, Italy; 4 Unit of Urology, Department of Surgery, Oncology and Gastroenterology, University of Padova, Padova, Italy

**Keywords:** Follicle stimulating hormone, Total motile sperm count, Dynamic ultrasound with contrast agent, Mean transit time

## Abstract

**Purpose:**

Varicocele is a recognized male factor of infertility. Surgical/microsurgical correction represents a therapeutical option to improve fertility outcomes but predictive parameters for the identification who can actually benefit from varicocele correction are under investigated. Here we aimed to identify baseline predictors of semen outcome improvement after varicocele treatment by scleroembolization approach.

**Methods:**

85 patients receiving varicocele treatment by anterograde scleroembolization (ASE, *N* = 42) or retrograde scleroembolization (RSE, *N* = 43) were retrospectively recruited. Basal and 6-months follow-up evaluation of semen, hormonal and ultrasound parameters (US) were performed to address the respective effect of varicocele treatment. In addition, basal parameters were assessed as clinical outcome predictors.

**Results:**

Varicocele grade reduction was observed in more than 90% of patients (*P* < 0.001). Compared to basal, significant increase of sperm concentration (10.0 ± 9.2 × 10^6^cells/mL vs. 23.4 ± 26.9 × 10^6^cells/mL; *P* < 0.001), total sperm count (TSC 39.0 ± 54.5 × 10^6^cells vs. 70.3 ± 90.6 × 10^6^cells, *P* < 0.001) and total motile sperm count (TMS, 9.8 ± 12.8 × 10^6^cells vs. 36.7 ± 58.1 × 10^6^cells, *P* < 0.001), was observed, with no differences between RSE or ASE. All US parameters were also improved (all *P* < 0.001). Logistic regression analysis of basal semen and ultrasound parameters showed that basal sperm motility and left testis-mean transit time (L-MTT) were associated with TSC and TMS doubling at follow-up. However, Receiver Operating Characteristic curve analysis showed that only basal L-MTT basal sperm was consistently associated with TSC and TMS doubling at follow-up (respectively: AUC = 0.834, CI: 0.747–0.921 and AUC = 0.805, CI: 0.708–0.901; both *P* < 0.001).

**Conclusion:**

Baseline sperm count and US parameters represent useful clinical descriptors to address those subjects eligible for varicocele correction.

**Supplementary Information:**

The online version contains supplementary material available at 10.1007/s40618-026-02844-0.

## Introduction

Varicocele is defined as an abnormal venous dilation within the pampiniform plexus of testis, mostly associated with venous reflux [1]. With a typical onset during puberty, varicocele is a common clinical condition in andrological practice, with an estimated prevalence of 15% within the general population. Since the prevalence of varicocele rises to 35–80% in infertile patients, it results as one of the most common reversible male factor of infertility (MFI) [2, 3]. However, current hypotheses suggest varicocele to be actually responsible for a lower proportion of MFI [4]. In approximately 90% of the cases varicocele is left-sided whilst, in the remaining 10%, is bilateral with a higher involvement of the left side [2]. The diagnosis of varicocele associates with major clinical outcomes such as left testicular hypotrophy, found in 20–35% of patients, altered testicular-endocrine function and qualitative/quantitative impairment of semen parameters, resulting in an overall reduction of fertility potential after the exclusion of other major causes of MFI [5, 6]. Different pathophysiological mechanisms have been invoked to explain the varicocele-associated testicular damage, from tissue hypoxia and oxidative stress to the increased scrotal temperature associated with reduced heat dissipation [7–9]. Accordingly, a recent study from our group highlighted a major role of the impairment of left testis microcirculation in varicocele-related issues [10]. In particular, using a contrast-enhanced ultrasound (US) approach, a significant correlation was found between left testis perfusion time, evaluated by the mean transit time (MTT) US parameter, and semen parameters. Importantly, an MTT value exceeding 36 s was proven to be an independent predictor of venous intratesticular stasis and oligozoospermia [10].

Varicocele correction by either surgical/microsurgical or endovascular approaches represents a therapeutical option to improve the fertility outcomes associated with this clinical condition. However, considering the different degrees of invasiveness of surgical approaches and the controversial results about the inconsistent improvement observed in testis function, a gold standard strategy is not yet defined. Current guidelines from the American Urological Association (AUA) and the European Urological Association (EAU) do not identify threshold values, within available semen and hormonal parameters, according to which referring for varicocele correction by microsurgical/endovascular treatment [11, 12]. In this frame, the possible predictive role of the novel vascular characterization of the testis by US in current basal assessment of varicocele, in addition to standard semen and hormonal parameters, is still poorly investigated. Furthermore, the clinical benefit associated with varicocele correction in patients with clear signs of primary testis disease, such as high serum follicle stimulating hormone (FSH), azoospermia or severe oligozoospermia, is currently debated [13]. Indeed, available data showed no major beneficial effect on azoospermia, cryptozoospermia or complete isolated severe asthenozoospermia upon varicocele correction, being largely related to other etiological factors such as genetics [14]. On these bases, a thorough clinical evaluation, including complete anamnesis, medical/biochemical/ultrasound evaluation and complete semen analysis, remains a key step to address the characterization of MFI in patients [15].

In this study we aimed to identify reliable predictors of semen outcome, by the evaluation of a group of patients affected by varicocele, undergone to scleroembolization and characterized for semen, hormonal and ultrasound parameters.

## Methods

### Study design and patients

This was a retrospective observational study approved by the Territorial Ethics Committee Central - Eastern Veneto Area (study code# AOP3137). The study complied with the guidelines of the Helsinki Declaration [16]. All patients provided a signed informed consent for the retrospective study participation and were recruited at the Unit of Andrology and Reproductive Medicine of the University Hospital of Padova (Italy).

The analysis involved patients aged 18–55 years attending the outpatient Unit in the period January 2009-August 2025. Inclusion criteria were: diagnosis of varicocele associated with primary infertility and total sperm count (TSC) ≤ 40 × 10^6^ cells/ejaculate according to WHO criteria [17] and adherence to the follow-up visit scheduled 6 months after treatment as per standard clinical management of varicocele. Exclusion criteria were: age ≤ 18 years or ≥ 55 years, other known causes of infertility, including with positive microbiological culture and/or semen detection of human papillomavirus (HPV), positive screening for human immunodeficiency virus (HIV), hepatitis C (HCV) or B (HBV) viruses, concurrent treatment with sex steroids or gonadotropins, diagnosis of severe neoplasms or psychiatric disorders, severe secondary pathologies that may interfere with the study results.

Patients were eligible for varicocele treatment according to current urological guidelines [12, 18]. The unbiased and virtually-random assignment of patients to either Tauber’s anterograde scleroembolization (ASE), performed at the Unit of Urology, University-Hospital of Padova (Padova, Italy). or retrograde scleroembolization (RSE), performed at the Unit of Radiology, University-Hospital of Padova (Padova, Italy), was addessed per the earliest convenience based on waiting lists as standard clinical practice [19, 20]. All patients were evaluated at basal, before varicocele treatment, and at six months from intervention. Only patients in which procedural ASE or RSE was successfully accomplished were considered for the analysis. Patients in which procedural or anatomical factors unabled scleroembolization procedures, were excluded. All patients provided a peripheral blood sample, obtained by 8:00 am on a fasting state, for the evaluation of serum FSH, LH, total testosterone (TT), estradiol (E2) and prolactin (PRL) by electrochemiluminescence immunoassay methods (Cobas^®^; Roche Diagnostics, Mannheim, Germany). Semen analyses, performed according to the WHO laboratory manual for examination and processing of human semen – editions from fourth to sixth, included: sperm concentration, total sperm count (TSC), motility (further distinguished in type a, progressive, type a + b, total progressive and non-progressive and type c, non-motile), viability, typical forms. In order to provide the harmonic evaluation of semen outcomes across the whole range of retrospective enrollment (January 2009-August 2025), semen parameters were considered only as raw continuous variables and eventually clustered into unbiased categorical variables as quartiles as described below. Total motile sperm (TMS) count TMS was a derived parameter obtained from the product of TSC × type a + b motility. All analyses were performed according to standard procedures at the Unit of Laboratory Medicine of the University Hospital of Padova. Semen evaluation was replicated two times both at basal and at follow-up and the mean value of replicates was considered for the analysis.

Sperm DNA fragmentation, chromatin integrity and aneuploidies assessment were performed by respectively: terminal deoxynucleotidyl transferase dUTP nick end labeling (TUNEL) test, acridine orange test and fluorescence in situ hybridization (FISH) tests as previously described [21].

Medical and pharmacological history of patients, including lifestyle habits, were extracted from medical records. Specifically, data included: dietary and smoking habits, alcohol consumption, physical activity, sexual habits, andrological evidence, such as trauma, infections and testicular surgeries, presence of rheumatological, renal, endocrinological, gastroenterological, neoplastic, psychiatric disorders, family history of testicular disorders and infertility and regular use of medications that may influence testicular function, such as sex steroids or gonadotropins. The andrological examination aimed to explore the structural and morphological characteristics of the penis, testicles, epididymis, vas deferens, spermatic cord structures, prostate and the assessment of secondary sexual characteristics. Particular importance was paid for the assessment of testicular volume by ultrasound analysis as detailed below. The physical examination was conducted in both supine and standing positions, both at rest and during a Valsalva maneuver. Patients missing at least one of clinical data considered were excluded from the analysis.

## Scrotal doppler ultrasound and testicular contrast harmonic imaging

Patients were evaluated at dynamic ultrasound with contrast agent (US, Philips Epiq Elite, Milan, Italy) by the same trained medical staff (P.P. and N.C.) at all time points. US was performed with the patient lying in the supine position and the penis lifted towards the abdomen. A high-resolution linear Doppler probe (5–18 MHz) was used to collect transverse and sagittal images of both testes, in order to define volume, parenchymal echotexture, and perfusion status. Images were acquired and interpreted only by trained and experienced medical personnel (P.P. and N.C.). The US evaluation was performed according to most recent recommendations on scrotal ultrasound [22]. Varicocele was categorized into a five degrees scale according to the recommendations or the Penile Imaging Working Group of the European Society of Urogenital Radiology (ESUR-SPIWG) and the European Academy of Andrology [23] (http://www.andrologyacademy.net/studies).

Testicular contrast harmonic imaging was performed by antecubital vein injection of 2.5 ml of a contrast agent composed of microbubbles of sulfur hexafluoride, stabilized with phospholipids, immediately followed by a rapid injection of 5 ml of 0.9% sodium chloride solution. A 3-minute video clip at 15 frames per second was recorded starting 20 s after the contrast agent injection. Video clips were automatically analyzed using the Qontrast software (Bracco, Milano, Italy) as previously described [24]. The software processed of the intensity-time curves in an operator-unbiased manner and calculated the time of arrival of the contrast agent in the arteriolar circulation (wash-in), the TTP, the time of arrival of the contrast agent in the venular circulation (wash-out), and MTT. The procedure was repeated for the contralateral testicle after a 10-minute interval to allow for complete elimination of the contrast agent.

### Statistical analysis

Data were collected in a pseudo-anonymized dataset on a computer device and all statistical analyses were performed using SPSS software version 23.0 (SPSS Inc, Chicago, USA). The results were expressed as means ± standard deviation (SD). The prevalence of semen, hormonal, and ultrasound alterations was calculated in percentage and the Chi-Square test was used to assess prevalence differences through contingency tables. The Kolmogorov–Smirnov test was used to verify normal distribution of continuous variables. Comparison of mean values of continuous variables between two groups was performed by Student’s *t*-test. Comparison between two or more groups showing non-normal distribution of continuous variables was performed by non-parametric Kruskal-Wallis test. Differences in the mean value of parameters between more than two groups was performed with the analysis of variance (ANOVA) test with Bonferroni’s post-hoc test for pairwise comparisons between subgroups. Multivariate analysis with Bonferroni’s post-hoc correction was used to evaluate seminal, hormonal, and ultrasound parameters upon patients’ stratification. The association between a set of continuous with TSC or total motile sperm (TMS) doubling from basal as dichotomous outcome, was evaluated by logistic regression analysis, and the respective odds ratios (ORs) with 95% confidence intervals (CIs) was calculated. Receiver operating characteristic (ROC) curve was calculated by plotting sensitivity values on the y axis and 1-sensitivity values on the x axis. The area under the curve (AUC) was determined and evaluated by Sweets classification and the cutoff values for each considered variable have been calculated with Youden’s S statistics. The significance level of p was set to 0.05.

## Results

The retrospective analysis between January 2009-August 2025 allowed the recruitment of 85 patients with diagnosis of varicocele, 42 of which underwent to anterograde scleroembolization (ASE) according to Tauber’s method whilst 43 underwent to retrograde scleroembolization (RSE). Clinical and demographic data of patients at basal, and further distinguished for scleroembolization approach, are reported in Table 1. The comparison of demographic, seminal, hormonal and US parameters showed no significant differences between RSE and ASE patients. Major differences among the two groups of patients were observed in regard of the varicocele grading according to ESUR-SPIWG classification, since grade III was more represented in RSE patients, whilst grade V was more represented in ASE patients (respectively: grade III 18/43 RSE vs. 8/42 ASE and grade V 7/42 ASE vs. 3/42 RES; *P* = 0.034 at χ^2^ test). Finally, we found a significant difference between left and right testis volume, being the left smaller respectively: left 11.8 ± 3.2 mL, right 14.5 ± 4.0 mL, *P* < 0.001).

**Table 1:** Basal clinical and demographic data of 85 male patients with varicocele, undergone to either Tauber’s anterograde scleroembolization (ASE) or retrograde scleroembolization (RSE) surgery

Parameter	Overall (*N* = 85)	ASE (*N* = 42)	RSE (*N* = 43)	*P* value (ASE vs. RSE)
Age (years)	27.0 ± 4.9	26.9 ± 4.6	27.2 ± 5.3	0.794
*Hormonal parameters*				
FSH (IU/mL)	7.7 ± 6.5	8.0 ± 8.6	7.5 ± 3.4	0.728
LH (IU/mL)	6.0 ± 3.3	6.2 ± 4.3	5.9 ± 1.9	0.625
TT (nmol/L)	15.5 ± 5.6	15.6 ± 5.4	15.3 ± 5.8	0.815
E2 (pmol/L)	77.2 ± 31.3	78.8 ± 34.5	75.7 ± 28.1	0.660
PRL (ng/mL)	14.5 ± 6.1	14.1 ± 5.6	14.9 ± 6.5	0.553
*Semen parameters*				
Semen volume (mL)	2.5 ± 1.4	2.3 ± 1.4	2.6 ± 1.3	0.303
Semen pH	7.5 ± 0.2	7.5 ± 0.2	7.5 ± 0.2	0.766
Sperm Concentration (10^6^ cells/mL)	10.0 ± 9.2	11.6 ± 11.2	8.4 ± 6.4	0.106
Total Sperm Count (10^6^ cells)	20.6 ± 15.9	19.3 ± 13.0	21.7 ± 18.4	0.484
Cells with type-a progressive motility (%)	34.2 ± 19.5	35.0 ± 19.4	33.4 ± 19.9	0.703
Cells with type-b non-progressive motility (%)	9.2 ± 6.4	9.5 ± 6.8	9.0 ± 6.0	0.707
Cells with type-a + b motility (%)	42.2 ± 19.9	43.2 ± 19.7	41.2 ± 20.3	0.647
Not-motile cells (%)	56.6 ± 20.0	55.5 ± 19.5	57.6 ± 20.7	0.623
Total motile sperm count (10^6^ cells/mL)	9.8 ± 12.8	8.9 ± 7.4	10.7 ± 16.5	0.508
Viable spermatozoa (%)	73.7 ± 11.7	75.4 ± 11.2	72.1 ± 12.1	0.194
Cells with typical morphology (%)	7.1 ± 5.0	7.5 ± 5.6	6.8 ± 4.3	0.532
Percentage of anti-sperm antibodies (N, %)	8, 12.1 ± 12.2	4, 19.0 ± 14.8	4, 5.3 ± 1.3	0.160
TUNEL (%)	85.4 ± 7.1	85.2 ± 7.3	85.5 ± 7.0	0.853
Acridine Orange (%)	84.0 ± 5.8	84.1 ± 5.9	84.0 ± 5.8	0.901
FISH 18,X, Y (%)	0.9 ± 0.3	1.0 ± 0.4	0.9 ± 0.3	0.239
FISH 18,13,21 (%)	1.1 ± 0.3	1.1 ± 0.3	1.1 ± 0.3	0.879
*Testis parameters*				
Volume-L (mL)	11.8 ± 3.2	11.6 ± 3.6	11.9 ± 2.8	0.680
Volume-R (mL)	14.5 ± 4.0	14.0 ± 4.0	14.9 ± 4.0	0.307
Parenchymal homogeneity-L				
Homogeneous (N; %)	49; 57.6%	28; 66.7%	21; 48.8%	0.178^a^
Inhomogeneous (N; %)	26; 30.6%	9; 21.4%	17; 39.6	
Severe Inhomogeneous (N; %)	10; 11.8%	5; 11.9%	5; 11.6%	
Parenchymal homogeneity-R				
Homogeneous (N; %)	68; 80.0%	35; 83.3%	33; 76.7%	0.454^a^
Inhomogeneous (N; %)	12; 14.1%	4; 9.5%	8; 18.6%	
Severe Inhomogeneous (N; %)	5; 5.9%	3; 7.2%	2; 4.7%	
Varicocele grade-L				
II (N; %)	5; 5.8%	**1; 2.4%**	**4; 9.3%**	**0.034** ^**a**^
III (N; %)	26; 30.6%	**8; 19.0%**	**18; 41.9%**	
IV (N; %)	44; 51.8%	**26; 61.9%**	**18; 41.9%**	
V (N; %)	10; 11.8%	**7; 16.7%**	**3; 7.0%**	
Varicocele grade-R				
0 (N; %)	76; 89.4%	39; 92.9%	37; 86.0%	0.336^a^
I (N; %)	7; 8.2%	3; 7.1%	4; 9.3%	
II (N; %)	2; 2.4%	0; 0%	2; 4.7%	
*Ultrasound parameters*				
TTP-L (s)	21.4 ± 4.3	21.4 ± 4.5	21.4 ± 4.2	0.982
MTT-L (s)	40.2 ± 8.2	41.0 ± 8.7	39.5 ± 7.6	0.401
Wash in-L (s)	13.7 ± 5.0	14.0 ± 6.0	13.5 ± 3.8	0.638
Wash out-L (s)	11.5 ± 3.5	11.3 ± 4.0	11.7 ± 2.9	0.659
TTP-R (s)	20.2 ± 5.4	19.7 ± 4.4	20.7 ± 6.2	0.442
MTT-R (s)	35.2 ± 6.4	35.4 ± 5.8	35.0 ± 7.1	0.761
Wash in-R (s)	29.1 ± 6.4	29.3 ± 5.9	28.9 ± 6.9	0.747
Wash out-R (s)	25.5 ± 6.5	25.7 ± 6.5	25.2 ± 6.5	0.695

FAS: Follicle stimulating hormone, LH: Luteinizing hormone, TT: Total testosterone, E2; Estradiol, PRL; Prolactin, TUNEL: Terminal deoxynucleotidyl transferase-mediated deoxyuridine (TdT)  triphosphate (dUTP) nick end labeling assay; FISH: Fluorescent in-situ hybridization; L: Left; R: Right, TTP: Time to peak, MTT: Mean transit timeAll data are reported as mean value ± standard deviation. Significant p values are in bold (^a^: p value at Pearson’s Chi-square test)

## Basal to follow-up evaluation of patients

Results of the basal vs. follow-up comparison of demographic, seminal, hormonal and US parameters of the whole study group is reported in Table 2. Independently form the scleroembolization approach, data from the overall study cohort showed that varicocele correction was associated with significant reduction of the grade of varicocele compared to basal (*P* < 0.001), with more than 90% of patients displaying a degree equal or lower than one at follow-up evaluation. In addition, the comparison of hormonal and semen parameters at follow-up vs. basal showed a significant reduction of FSH levels together with the increase of semen volume, sperm concentration, TSC, percentage of motile spermatozoa, TMS, and cell viability. Interestingly, also second level parameters showed an improvement since the percentage of TUNEL-negative and acridine orange-negative cells were significantly increased at follow-up. In addition, the percentage of cells bearing chromosomal aneuploidies, both for sexual ad for autosomal chromosome, were reduced. On the other hand, varicocele correction was associated with a highly significant reduction of all US parameters, indicative of an improved testis blood flow.

**Table 2:** Clinical and demographic data, at basal and follow-up, of 85 male patients with varicocele, undergone to either Tauber’s anterograde scleroembolization (ASE) or retrograde scleroembolization (RSE) surgery

Parameter	Overall (*N* = 85)			ASE (*N* = 42)			RSE (*N* = 43)			*P* value
	BASAL	FOLLOW-UP	*P* value	BASAL	FOLLOW-UP	*P* value	BASAL	FOLLOW-UP	*P* value	
*Hormonal parameters*										
FSH (IU/mL)	**7.7 ± 6.5**	**7.3 ± 5.2**	**0.028**	8.0 ± 8.6	7.3 ± 6.7	0.646	7.5 ± 3.4	7.3 ± 3.2	0.852	0.969
LH (IU/mL)	6.0 ± 3.3	6.0 ± 3.1	0.901	6.2 ± 4.3	6.2 ± 4.0	0.997	5.9 ± 1.9	5.9 ± 2.1	0.942	0.575
TT (nmol/L)	15.5 ± 5.6	16.1 ± 4.6	0.186	15.6 ± 5.4	16.2 ± 4.3	0.497	15.3 ± 5.8	16.1 ± 4.8	0.469	0.949
E2 (pmol/L)	77.2 ± 31.3	78.5 ± 25.8	0.631	78.8 ± 34.5	77.7 ± 26.0	0.937	75.7 ± 28.1	79.3 ± 26.2	0.567	0.795
PRL (ng/mL)	14.5 ± 6.1	13.9 ± 5.4	0.169	14.1 ± 5.6	13.2 ± 5.0	0.439	14.9 ± 6.5	14.6 ± 5.8	0.840	0.266
*Semen parameters*										
Semen volume (mL)	**2.5 ± 1.4**	**3.0 ± 1.6**	**< 0.001**	**2.3 ± 1.4**	**3.0 ± 1.8**	**0.024**	2.6 ± 1.3	3.0 ± 1.3	0.279	0.944
Semen pH	7.5 ± 0.2	7.5 ± 0.2	0.960	7.5 ± 0.2	7.5 ± 0.2	0.948	7.5 ± 0.2	7.5 ± 0.2	0.957	0.739
Sperm Concentration (10^6^ cells/mL)	**10.0 ± 9.2**	**23.4 ± 26.9**	**< 0.001**	**11.6 ± 11.2**	**23.0 ± 21.9**	**0.013**	**8.4 ± 6.4**	**24.1 ± 31.6**	**< 0.001**	0.813
Total Sperm Count (10^6^ cells)	**39.0 ± 54.5**	**70.3 ± 90.6**	**< 0.001**	**19.3 ± 13.0**	**64.4 ± 91.8**	**0.001**	**21.7 ± 18.4**	**63.3 ± 70.2**	**0.001**	0.939
Cells with type-a progressive motility (%)	**34.2 ± 19.5**	**37.7 ± 19.3**	**0.030**	35.0 ± 19.4	39.7 ± 18.3	0.309	33.4 ± 19.9	36.1 ± 20.3	0.517	0.403
Cells with type-b non-progressive motility (%)	9.2 ± 6.4	9.4 ± 6.5	0.795	9.5 ± 6.8	9.8 ± 7.4	0.929	9.0 ± 6.0	9.1 ± 5.7	0.906	0.663
Cells with type-a + b motility (%)	**42.2 ± 19.9**	**47.1 ± 20.5**	**0.009**	43.2 ± 19.7	49.4 ± 19.5	0.201	41.2 ± 20.3	45.4 ± 21.5	0.346	0.370
Not-motile cells (%)	53.6 ± 21.2	47.3 ± 19.2	0.053	55.5 ± 19.5	50.8 ± 19.8	0.344	57.6 ± 20.7	54.8 ± 21.7	0.530	0.363
Total motile sperm count (10^6^ cells/mL)	**9.8 ± 12.8**	**36.7 ± 58.1**	**< 0.001**	**8.9 ± 7.4**	**38.0 ± 62.2**	**0.002**	**10.7 ± 16.5**	**36.2 ± 54.9**	**0.006**	0.839
Viable spermatozoa (%)	**73.7 ± 11.7**	**77.3 ± 11.5**	**< 0.001**	75.4 ± 11.2	77.8 ± 10.9	0.476	72.1 ± 12.1	76.6 ± 12.3	0.071	0.637
Cells with typical morphology (%)	7.1 ± 5.0	7.6 ± 4.1	0.348	7.5 ± 5.6	7.8 ± 4.6	0.806	6.8 ± 4.3	7.4 ± 3.6	0.571	0.653
Percentage of anti-sperm antibodies (N, %)	8, 12.1 ± 12.2	8, 16.6 ± 12.0	0.080	4, 19.0 ± 14.8	4, 18.9 ± 12.7	0.988	4, 5.3 ± 1.3	4, 7.5 ± 3.0	0.774	0.095
TUNEL^−^ cells (%)	**85.4 ± 7.1**	**89.4 ± 5.9**	**< 0.001**	**85.2 ± 7.3**	**89.4 ± 6.1**	**0.008**	**85.5 ± 7.0**	**89.6 ± 5.8**	**0.005**	0.890
Acridine Orange^−^ cells (%)	**84.0 ± 5.8**	**88.7 ± 6.3**	**< 0.001**	**84.1 ± 5.9**	**89.0 ± 6.3**	**< 0.001**	**84.0 ± 5.8**	**88.3 ± 6.3**	**0.001**	0.585
FISH 18,X, Y (%)	**0.95 ± 0.35**	**0.85 ± 0.25**	**0.022**	0.97 ± 0.37	0.87 ± 0.28	0.124	0.90 ± 0.30	0.83 ± 0.21	0.286	0.560
FISH 18,13,21 (%)	**1.10 ± 0.28**	**0.91 ± 0.25**	**< 0.001**	**1.09 ± 0.30**	**0.89 ± 0.27**	**< 0.001**	**1.11 ± 0.27**	**0.94 ± 0.23**	**0.003**	0.395
*Testis parameters*										
Volume L (mL)	**11.8 ± 3.2**	**11.9 ± 3.2**	**0.033**	11.6 ± 3.6	11.8 ± 3.7	0.991	11.9 ± 2.8	12.0 ± 2.7	0.901	0.741
Volume R (mL)	14.5 ± 4.0	14.5 ± 3.8	0.701	14.0 ± 4.0	14.1 ± 3.9	0.942	14.9 ± 4.0	14.9 ± 3.7	0.991	0.355
Varicocele grade L										
0 (N. %)	**0; 0%**	**67; 78.8%**	**< 0.001** ^**a**^	**0; 0.0%**	**34; 80.9%**	**< 0.001** ^**a**^	**0; 0%**	**33; 76.7%**	**< 0.001** ^**a**^	0.175^a^
I (N. %)	**0; 0%**	**12; 14.1%**		**0; 0.0%**	**5; 11.9%**		**0; 0%**	**7; 16.4%**		
II (N; %)	**5; 5.9%**	**3; 3.5%**		**1; 2.4%**	**3; 7.2%**		**4; 9.3%**	**0; 0%**		
III (N; %)	**26; 30.6%**	**2; 2.3%**		**8; 19.0%**	**0; 0%**		**18; 41.9%**	**2; 4.6%**		
IV (N; %)	**44; 51.8%**	**1; 1.2%**		**26; 61.9%**	**0; 0%**		**18; 41.9%**	**1; 2.3%**		
V (N; %)	**10; 11.7%**	**0; 0%**		**7; 16.7%**	**0; 0%**		**3; 7.0%**	**0; 0%**		
*Ultrasound parameters*										
TTP-L (s)	**21.4 ± 4.3**	**16.9 ± 2.8**	**< 0.001**	**21.4 ± 4.5**	**16.7 ± 3.0**	**< 0.001**	**21.4 ± 4.2**	**17.0 ± 2.7**	**< 0.001**	0.681
MTT-L (s)	**40.2 ± 8.2**	**29.0 ± 4.2**	**< 0.001**	**41.0 ± 8.7**	**28.4 ± 4.3**	**< 0.001**	**39.5 ± 7.6**	**29.5 ± 4.1**	**< 0.001**	0.473
Wash in-L (s)	**13.7 ± 5.0**	**9.5 ± 2.2**	**< 0.001**	**14.0 ± 6.0**	**9.2 ± 2.1**	**< 0.001**	**13.5 ± 3.8**	**9.8 ± 2.3**	**< 0.001**	0.447
Wash out-L (s)	**11.5 ± 3.5**	**8.7 ± 2.1**	**< 0.001**	**11.3 ± 4.0**	**9.9 ± 3.4**	**< 0.001**	**11.7 ± 2.9**	**10.3 ± 3.0**	**< 0.001**	0.589
TTP-R (s)	**20.2 ± 5.4**	**16.2 ± 3.3**	**< 0.001**	**19.7 ± 4.4**	**16.0 ± 3.0**	**< 0.001**	**20.7 ± 6.2**	**16.5 ± 3.7**	**< 0.001**	0.635
MTT-R (s)	**35.2 ± 6.4**	**27.3 ± 4.2**	**< 0.001**	**35.4 ± 5.8**	**27.1 ± 4.3**	**< 0.001**	**35.0 ± 7.1**	**27.5 ± 4.1**	**< 0.001**	0.766
Wash in-R (s)	**29.1 ± 6.4**	**20.9 ± 4.2**	**< 0.001**	**29.3 ± 5.9**	**21.0 ± 4.7**	**< 0.001**	**28.9 ± 6.9**	**20.7 ± 3.8**	**< 0.001**	0.783
Wash out-R (s)	**25.5 ± 6.5**	**21.3 ± 4.2**	**< 0.001**	**25.7 ± 6.5**	**21.5 ± 4.6**	**0.001**	**25.2 ± 6.5**	**21.0 ± 3.8**	**0.001**	0.655

Abbreviations: Follicle stimulating hormone, FSH; luteinizing hormone, LH; total testosterone, TT; estradiol, E2; prolactin, PRL; TUNEL, terminal deoxynucleotidyl transferase-mediated deoxyuridine (TdT) triphosphate (dUTP) nick end labeling assay; FISH, Fluorescent in-situ hybridization; left, L; right, R; time to peak, TTP; mean transit time, MTT

All data are reported as mean value ± standard deviation. Significant p values are in bold (^a^: p value at Pearson’s χ-square test)

**Table 3:** Logistic regression analysis of basal semen (A) and ultrasound (B) parameters predicting total sperm count or total motile sperm doubling at follow-up

A	Output variable: Total Sperm Count Doubling		
Parameter	*P* Value	Odd Ratio	95% C.I.
Age	0.317	1.052	0.952–1.162
FSH	0.106	0.882	0.758–1.027
TT	0.845	0.991	0.905–1.085
Total Sperm Count	0.108	0.956	0.905–1.010
**Type A Motility**	**0.033**	**1.102**	**1.008–1.205**
TMS	0.273	1.054	0.960–1.157
Viability	0.835	1.005	0.959–1.053
Typical Morphology	0.761	0.985	0.891–1.088
	**Output variable: Total Motile Sperm Doubling**		
**Parameter**	**P Value**	**Odd Ratio**	**95% C.I.**
Age	**0.656**	**0.978**	**0.887–1.079**
FSH	0.181	0.910	0.792–1.045
TT	0.762	0.986	0.902–1.078
Total Sperm Count	0.991	0.991	0.957–1.026
**Type A Motility**	**0.047**	**1.092**	**1.001–1.190**
TMS	0.705	0.989	0.935–1.046
Viability	0.728	0.992	0.946–1.039
Typical Morphology	0.504	0.967	0.877–1.066
**B**	**Output variable: Total Sperm Count Doubling**		
**Parameter**	**P Value**	**Odd Ratio**	**95% C.I.**
L-TTP	0.720	1.043	0.828–1.315
R-TTP	0.215	1.151	0.921–1.437
**L-MTT**	**0.002**	**1.183**	**1.061–1.319**
R-MTT	0.182	1.097	0.957–1.258
L-Wash IN	0.374	0.941	0.823–1.076
R-Wash IN	0.374	0.941	0.823–1.076
L-Wash OUT	0.189	1.066	0.969–1.173
R-Wash OUT	0.347	0.951	0.857–1.056
	**Output variable: Total Motile Sperm Doubling**		
**Parameter**	**P Value**	**Odd Ratio**	**95% C.I.**
L-TTP	0.488	1.085	0.861–1.387
R-TTP	0.598	1.057	0.861–1.298
**L-MTT**	**0.022**	**1.125**	**1.017–1.243**
R-MTT	0.290	1.071	0.943–1.217
L-Wash IN	0.206	0.912	0.792–1.052
R-Wash IN	0.632	1.049	0.863–1.275
**L-Wash OUT**	**0.019**	**1.129**	**1.020–1.249**
R-Wash OUT	0.651	0.978	0.886–1.079

Abbreviations: Follicle stimulating hormone, FSH; total testosterone, TT; total motile sperm count, TMS; left, L; right, R; time to peak, TTP; mean transit time, MTT; confidence interval, C.I

Significant associations are in bold

All the aforementioned basal vs. follow-up variations of hormonal, seminal and US parameters were maintained in both ASE and RSE patients upon stratification by scleroembolization technique, exception made for semen volume in RSE patients which was unvaried at follow-up.

Importantly, no significant difference was observed between ASE and RSE patients for clinical parameters at follow-up, allowing to perform subsequent analyses on all patients as a whole group.

**Fig. 1:**
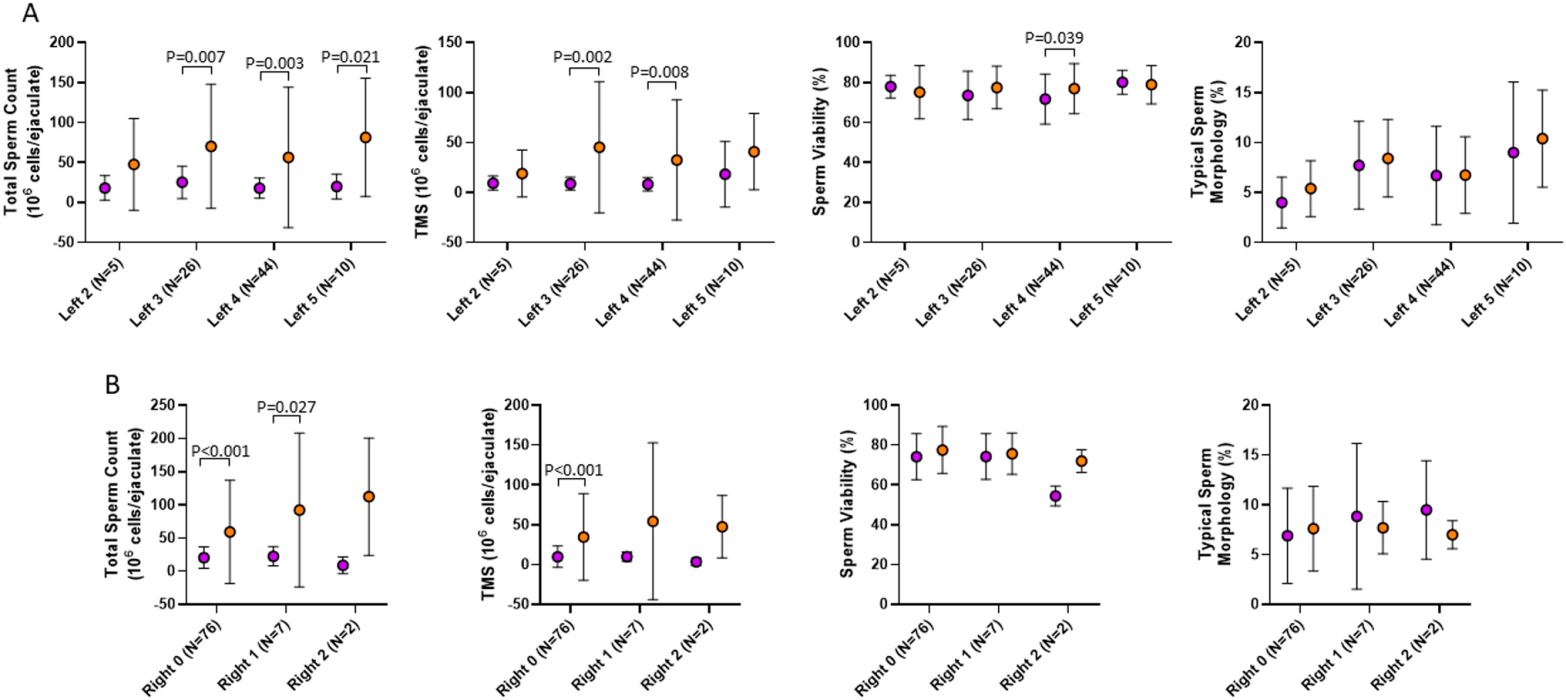
Multivariate analysis of basal-to-follow-up comparison of total sperm count and total motile sperm count (TMS) in 85 patients undergone to varicocele scleroembolization treatment, stratified according to basal left (**A**) and right (**B**) varicocele degree score according to the Penile Imaging Working Group of the European Society of Urogenital Radiology (ESUR-SPIWG) and the European Academy of Andrology [23]. Significance: comparison P values between conditions are indicated

In order to address the possible prognostic value of demographic and clinical parameters at basal, in terms of improvement of sperm parameters associated with the treatment, patients were preliminarily stratified according to left and right testis varicocele degree scale (Fig. 1A, B). Basal left testis varicocele degree treater than 2 was associated with a significant increase of the TSC at follow-up, whilst the TMS showed an increase only for degrees 2 and 3. Sperm viability showed some significant improvements only for patients with degree 3. On the other hand, basal right testis varicocele degree 0 and 1 were associated with a significant increase of the TSC. An increase of TMS was observed only for degree 0.

**Fig. 2:**
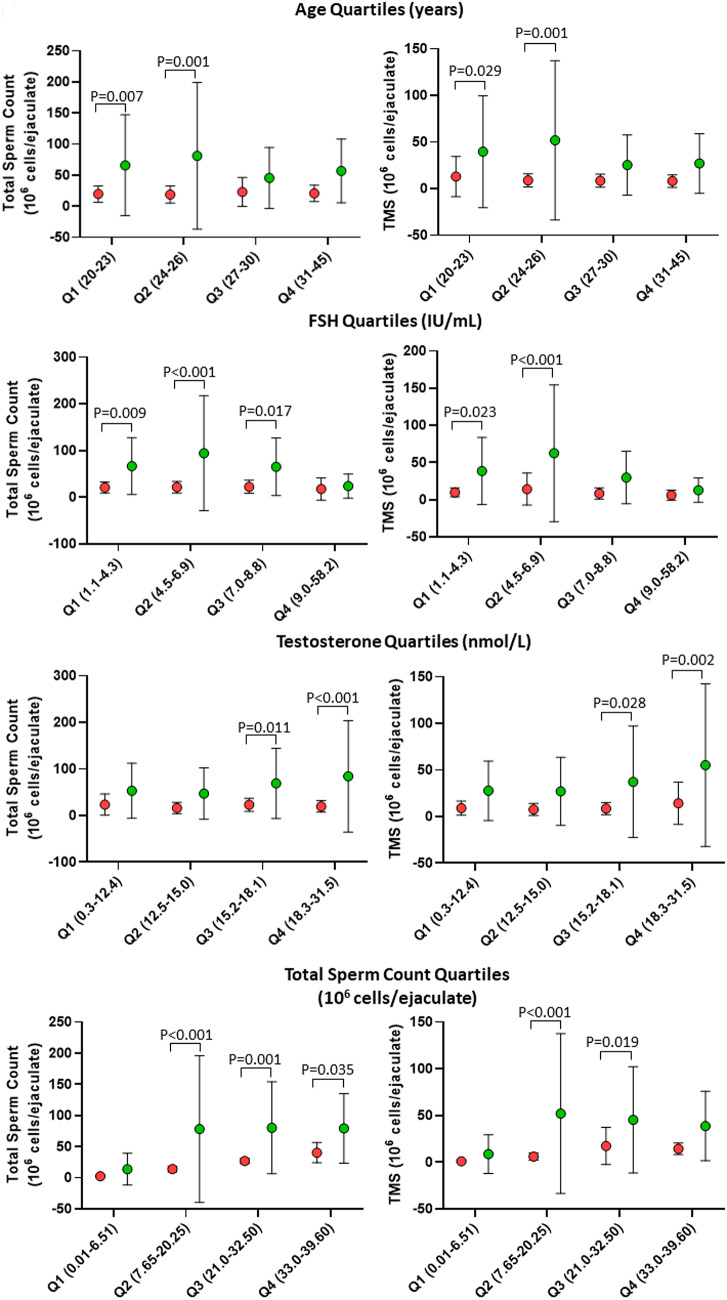
Multivariate analysis of basal-to-follow-up comparison of total sperm count and total motile sperm count (TMS) in 85 patients undergone to varicocele scleroembolization treatment, stratified according to basal quartiles of age, follicle stimulating hormone (FSH), total testosterone and total sperm count values. Significance: comparison P values between conditions are indicated

**Fig. 3:**
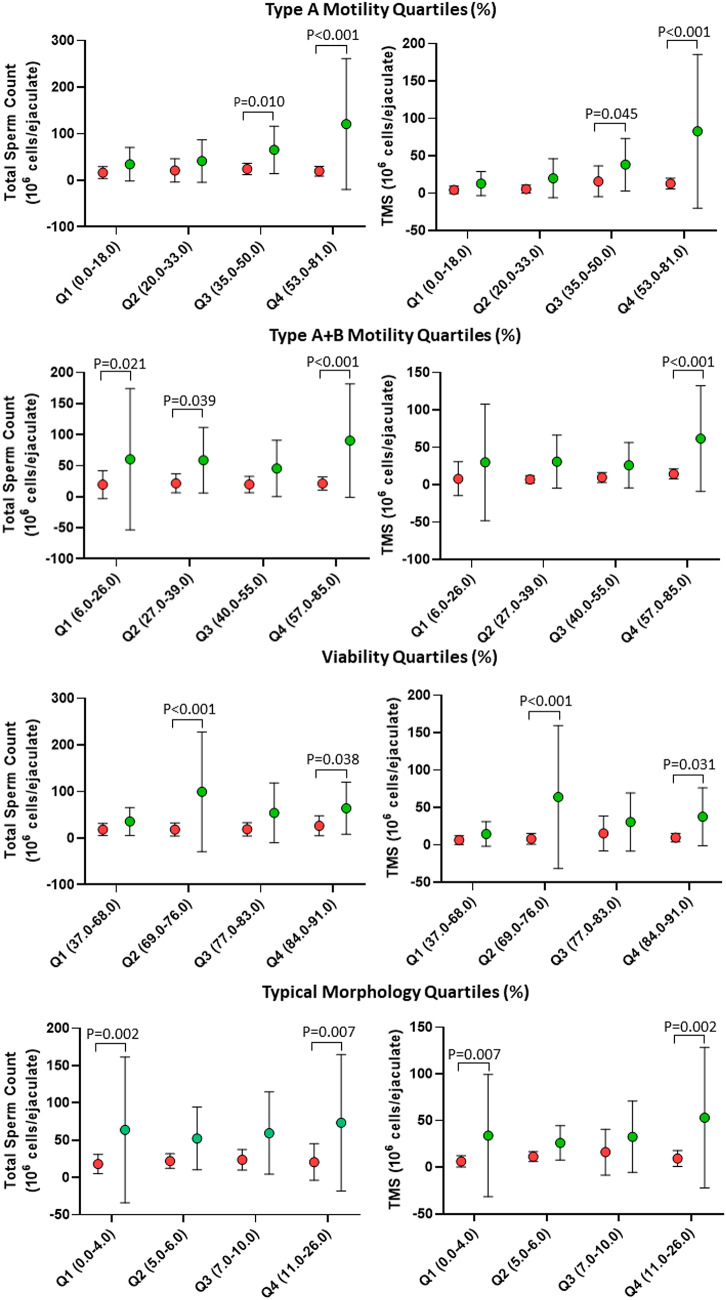
Multivariate analysis of basal-to-follow-up comparison of total sperm count and total motile sperm count (TMS) in 85 patients undergone to varicocele scleroembolization treatment, stratified according to basal quartiles of type A sperm motility, type A + B sperm motility, sperm viability and tipycoal sperm morphology. Significance: comparison P values between conditions are indicated

Patients were then stratified and according to quartiles of increasing basal age, FSH, total testosterone (TT), total sperm count, type A motility and typical morphology and evaluated for the basal-to-follow-up variation of TSC and TMS (Figs. 2 and 3). Varicocele correction was associated with a significant improvement of both TSC and TMS in patients relying to quartiles 1–2 of age, quartiles 3–4 of TT, quartiles 3–4 of type A motility, quartiles 2 and 4 of viability and quartiles 1 and 4 of typical sperm morphology. On the other hand, basal vs. follow-up TSC was significantly increased in relaying to quartiles 1–3 of FSH, whilst TMS was increased only for quartiles 1 and 2. Also patients relying to basal TSC quartiles 2–4 showed a significant increase of TSC at follow up, whilst TMS was increased only for quartiles 2–3. Finally, patients with basal type A + B motility relying to quartiles 1, 2 and 4 showed a significant increase of TSC, whilst an increase of TMS was observed only for quartile 4. Minor impact was observed on sperm viability and typical sperm morphology (Supplemental Table S1A). In particular, a significant increase of cell viability was observed for quartile 3 of basal type A motility, quartile 3 of basal type A + B motility and quartiles 1–2 of basal sperm viability. Differently a significant increase of typical sperm morphology was observed for basal quartiles 1 and 4 of typical sperm morphology.

**Fig. 4:**
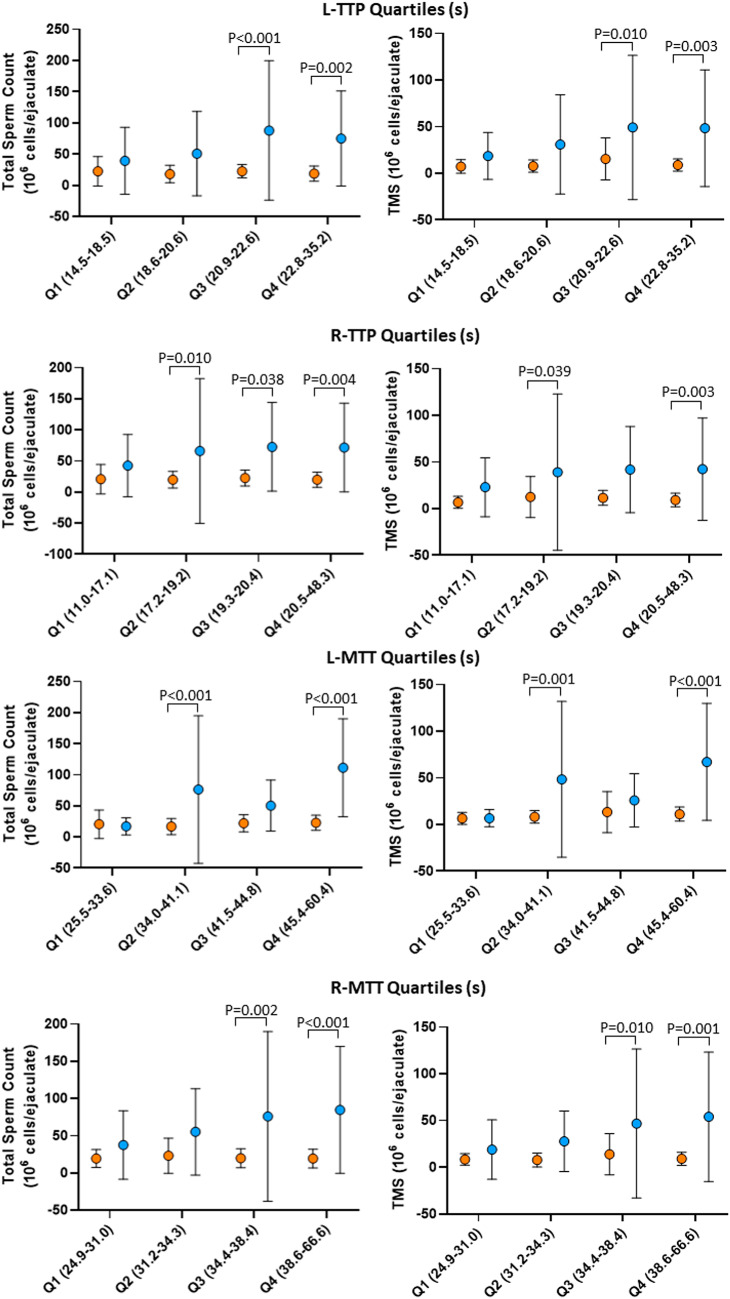
Multivariate analysis of basal-to-follow-up comparison of total sperm count and total motile sperm count (TMS) in 85 patients undergone to varicocele scleroembolization treatment, stratified according to basal quartiles of ultrasound left (L) and right (R) time to peak (TTP) and mean transit time (MTT). Significance: comparison P values between conditions are indicated

**Fig. 5:**
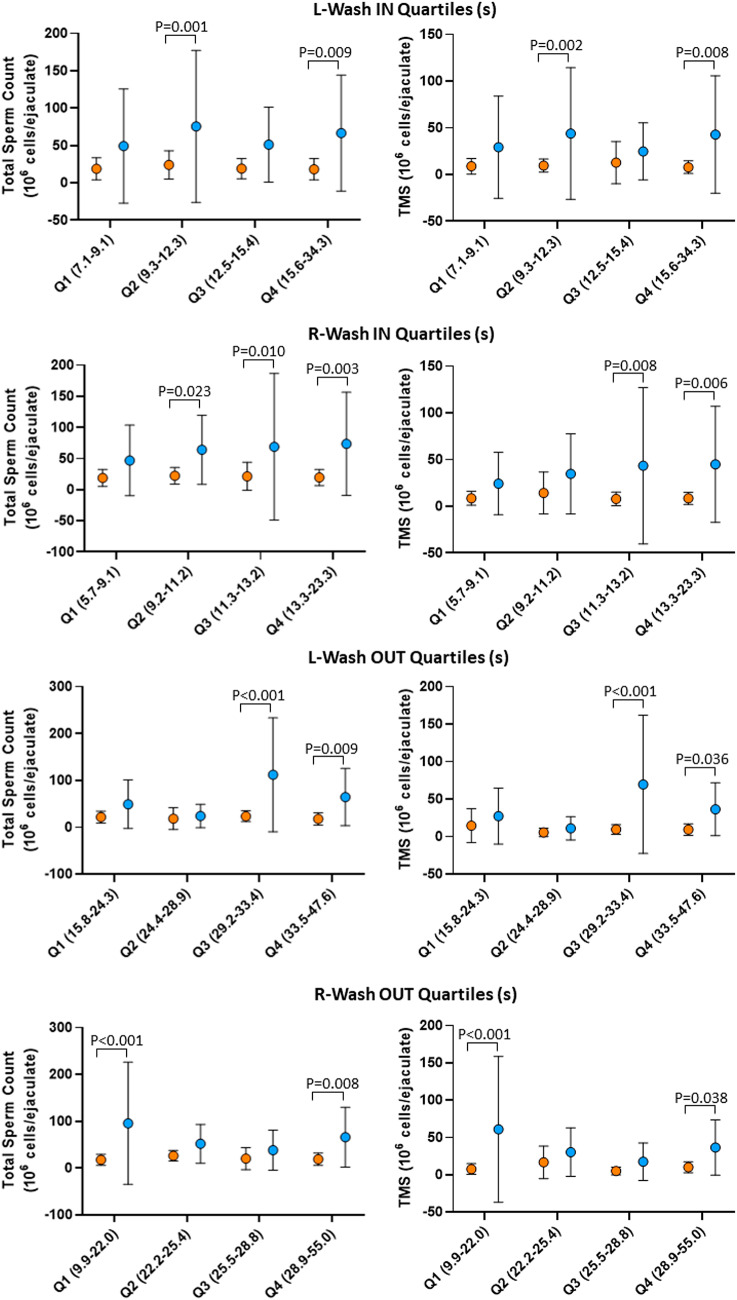
Multivariate analysis of basal-to-follow-up comparison of total sperm count and total motile sperm count (TMS) in 85 patients undergone to varicocele scleroembolization treatment, stratified according to basal quartiles of ultrasound left (L) and right (R) wash in time and wash out time. Significance: comparison P values between conditions are indicated

A similar approach was applied to basal US parameters, for which higher consistency between TSC and TMS was observed (Figs. 4 and 5). In fact, a significant increase of basal vs. follow-up TSC and TMS emerged for patients relying in quartiles 3–4 of L-TTP, quartiles 2 and 4 of L-MTT, quartiles 3–4 of R-MTT, quartiles 2 and 4 of L-Wash IN, quartiles 3–4 of L-Wash OUT and quartiles 1 and 4 of R-Wash OUT. Only basal R-Wash IN parameter showed some difference between the two outcome parameters since patients relying in quartiles 2, 3 and 4 showed a significant increase of TSC whilst an increase of TMS was observed only for quartiles 3–4. On the other hand, a significant increase of the basal-to-follow-up sperm viability was observed for basal quartile 4 of TTP-R, quartile 4 of TTP-L and quartile 3 of MTT-L (Supplemental Table S1B).

To better evaluate the association between basal pattern quartiles and TSC and TMS, a logistic regression analysis was performed to estimate the relative impact of basal age, FSH, TT, TSC, type A Motility, TMS, sperm viability and typical morphology, as continuous variables, in associating with TSC or TMS doubling at follow-up as dichotomous variable (Table 3A). Interestingly, basal type A motility showed an independent and significant association with both TSC and TMS doubling at follow-up (respectively, *P* = 0.033, OR = 1.102, 95% C.I 1.008–1.205 and *P* = 0.047, OR = 1.092, 95% C.I 1.001–1.190). A similar approach was then applied to basal US parameters as continuous variables (Table 3B), showing that L-MTT was independently and significantly associated with both TSC and TMS doubling at follow-up (respectively: *P* = 0.002, OR = 1.183, 95% C.I. 1.061–1.319 and *P* = 0.022, OR = 1.125, 95% C.I.1.017–1.243) whilst L-Wash out was associated with TMS doubling (*P* = 0.019, OR = 1.129, 95% C.I 1.020–1.249). Of note, neither scleroebolization procedures nor left or right varicocele grading was associated with either of the seminal outcome parameters (Supplemental Table S2A-B).

**Fig. 6:**
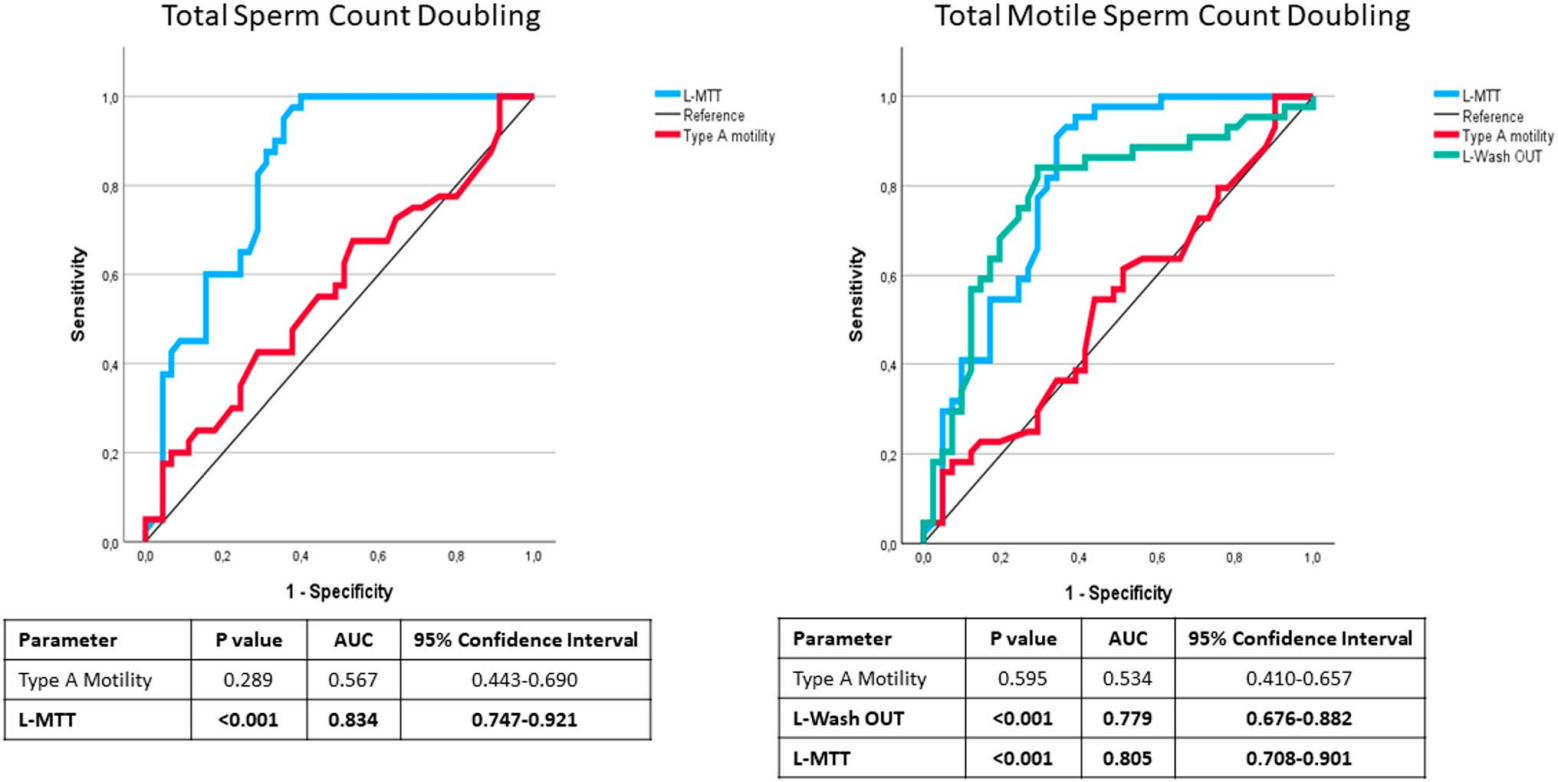
Receiver Operating Characteristic curve analysis of the association between basal type A sperm motility, left ultrasound mean transit time (L-MTT) and left ultrasound wash out time with tolat sperm count or total motile sperm count doubling after varicocele correction by scleroembolization technique

On this base, a receiver operating characteristic (ROC) analysis was performed in order to correlate type A motility and L-MTT as continuous variables with TSC doubling at follow-up and A motility, L-MTT and L-wash-out with TMS doubling (Fig. 6A, B). Only L-MTT values equal or greater than, respectively 35.95 s and 37.5 s were prognostic of TSC and TMS doubling at follow-up (respectively: *p* < 0.001, AUC = 0.834, 95% C.I. 0.747–0.921 and *P* < 0.001, AUC = 0.805, 95% C.I. 0.708–0.901). On the other hand, L-Wash OUT values equal or greater than 27.2 s were prognostic of TMS doubling (*P* < 0.001, AUC = 0.779, 95% C.I. 0.676–0.882).

## Discussion

In this study we provide evidence that the treatment of varicocele with scleroembolization procedure upon appropriate patient selection, associates with a significant improvement of semen parameters and bilateral testicular microcirculation status. In addition, basal US-based testicular contrast harmonic imaging assessment showed significant prognostic value to address those patients who would benefit most from varicocele correction in terms of total sperm count and/or total motile count increase.

Available data on the possible improvement of testis function upon varicocele correction shows controversial results. Nilsson et al. reported no significant improvement of semen parameters after surgical correction compared to controls [25]. Differently, Madgar et al. demonstrated marked improvements in sperm parameters and fertility rates [26]. On the other hand, recent studies further support the beneficial effects of varicocele treatment on semen quality, since Morini et al. observed significant improvements in sperm concentration and motility, along with a decrease in sperm head and tail abnormalities after surgical correction of varicocele [27]. Similarly, Mongioì et al. found significant improvements in seminal parameters and, importantly, showed that sclerotherapy led to enhanced spontaneous pregnancy rates compared to surgical varicocelectomy [28].

In spite of these evidence, major efforts are still required to address which basal parameters are prognostic for the indication of varicocele correction aimed at improving seminal outcome. Ory et al. showed that elevated FSH levels at basal are associated with poorer semen outcomes, emphasizing the importance of including hormonal assessments in the preoperative evaluation of patients with varicocele [29]. In addition, recent metanalyses showed that the preoperative FSH levels and luteinizing hormone (LH)/T ratio are promising prognostic parameters to a favorable outcome after varicocele treatment [30–32]. In this study we showed that, by the use of a multivariate analysis performed on quartiles of basal demographic and clinical parameters, some significant and consistent increase of TSC and/or TMS were obtained in younger patients, with basal age < 26 years, together with FSH < 8.8 IU/mL, total T > 15.2 nmol/L and TSC > 7.65🞨10^6^ cells/ejaculate. However, all these parameters failed to associate with the doubling TSC or TMS at follow-up, compared to basal, at logistic regression analysis. On the other hand, a consistent association with both TSC and TMS doubling was observed for basal L-MTT at both logistic regression and ROC analysis. In particular, a significant association with TSC and TMS doubling was observed for L-MTT values greater than, respectively, 35.95 s and 37.5 s. Strikingly, these threshold values are closed to those previously identified by Caretta et al. as predictors of low sperm count (TSC < 39🞨10^6^ cells/ejaculate) in varicocele patients [10]. Taken together, these data highlight the role of first and second level-diagnostic work-up of patients considered eligible for varicocele treatment, in order to likely exclude other causes of alteration of semen parameters. In fact, it is recognized that severe oligozoospermia and very high levels of FSH are associated with other causes of primary testis impairment than varicocele that would poorly benefit from the treatment [33, 34].

These findings reinforce the concept that the functional status of the testicular microcirculation represents a key determinant of spermatogenic efficiency. Recent data highlight that testicular hypoxia, a recognized consequence of impaired venous drainage in varicocele, may exert multifaceted deleterious effects on germ-cell metabolism, oxidative stress, epididymal maturation and spermatogenesis [35, 36]. In particular, the review by Lord et al. highlight how hypoxia can alter gene-expression, endocrine signalling and epididymal epithelial cell function in humans and animal models, thus providing a plausible explanation for the impairment of local micro-circulation that we observed as delayed MTT at US analysis. In this frame, the prognostic significance of delayed L-MTT may reflect an underlying hypoxic microenvironment, which becomes amenable to correction by the varicocele treatment.

The correction of venous stasis is then suggested to restore physiological perfusion gradients, oxygenation, and nutrient delivery to the seminiferous epithelium, promoting a functional recovery of the germinal line.

Beyond classical semen and hormonal parameters, integrating multiple indices could allow a more precise and individualized assessment of testicular function. A multimodal prognostic model, combining hormonal, biochemical, and imaging-derived parameters, could improve the selection of candidates for varicocele treatment and provide deeper insight into its physiological basis [34]. In practice, this approach could stratify patients according to their residual testicular reserve, helping to identify those more likely to benefit from treatment. Moreover, it could provide a conceptual framework linking imaging-derived microcirculatory findings with molecular and cellular mechanisms, including hypoxia and oxidative stress.

We acknowledge several limitations for this study. Firstly, the retrospective nature of the analysis and the relatively small sample size may limit the generalizability of our findings. We also excluded patients featured by unsuccessful scleroembolization procedures, thus missing to evaluate the reasons for failure that may provide additional insights, particularly with regard to technical aspects and procedural feasibility. In addition, we adopted TSC and TMS doubling to measure the semen outcome of varicocele treatment. As per standard practice, patient’s data on fertility outcomes were not available and data on seminal outcomes were used as valuable surrogates. In addition, whilst possibly questionable, the use of TSC and TMS doubling as endpoints is consistent with previous studies on this topic and can be considered clinically sound in a real-life scenario in order to provide the patient with a realistic perspective of the possible improvement of seminal parameters during pre-interventional counseling [37–40].

## Conclusions

In this study we showed that in well selected patients varicocele scleroembolization has beneficial effects on semen outcome and testis vascularization at ultrasound analysis. Although the overall characterization of the patient remains a key point for the eligibility for treatment, some parameters such as baseline sperm count and reduced testicular perfusion represent important clinical descriptors for the identification those subjects who can benefit most from the treatment.

## Supplementary Information

Below is the link to the electronic supplementary material.

Supplementary Material 1

Supplementary Material 2

## Data Availability

The data that support the findings of this study are available from the Padova University-Hospital but restrictions apply to the availability of these data, which were used under license for the current study, and so are not publicly available. Data are however available from the authors upon reasonable request and with permission of the Padova University-Hospital. For any information, contact luca.detoni@unipd.it.
