# Supplementary material for: Predictive parameters of semen outcome improvement after varicocele treatment by scleroembolization: a retrospective study
Source: J Endocrinol Invest. 2026 Apr 29;49(7):1565–80. doi: 10.1007/s40618-026-02844-0 (PMC13451323; doi:10.1007/s40618-026-02844-0)
Supplement: Supplementary file 1 [file 40618_2026_2844_MOESM1_ESM.docx]

**Supplemental Table S1**

**A** Baseline to follow up variations in sperm viability and morphology, according to distribution quartiles of basal demographic, hormonal and semen parameters in 85 patients undergone to varicocele treatment.

| **Basal parameter** | **Sperm Viability (%)** | | **P value** | **Typical Sperm Morphology (%)** | | **P value** |
| --- | --- | --- | --- | --- | --- | --- |
| **Age (years)** | **Basal** | **Follow up** |  | **Basal** | **Follow up** |  |
| **Q1 (20-23)** | 75.4±13.5 | 78.9±13.5 | 0.296 | 7.1±5.7 | 8.3±4.9 | 0.374 |
| **Q2 (24-26)** | 72.6±9.9 | 78.9±10.6 | 0.077 | 9.1±5.7 | 7.9±4.2 | 0.371 |
| **Q3 (27-30)** | 75.4±11.3 | 76.6±9.7 | 0.750 | 5.7±3.5 | 6.9±3.6 | 0.412 |
| **Q4 (31-45)** | 71.4±11.9 | 74.3±11.7 | 0.442 | 6.3±4.0 | 7.6±4.1 | 0.554 |
|  |  |  |  |  |  |  |
| **FSH (IU/mL))** | **Basal** | **Follow up** |  | **Basal** | **Follow up** |  |
| **Q1 (1.1-4.3)** | 77.8±9.2 | 81.5±9.0 | 0.273 | 5.9±3.5 | 7.4±4.3 | 0.276 |
| **Q2 (4.5-6.9)** | 77.2±12.4 | 80.7±10.2 | 0.283 | 8.9±5.6 | 8.7±4.0 | 0.871 |
| **Q3 (7.0-8.8)** | 66.9±11.8 | 70.1±12.8 | 0.364 | 5.9±4.1 | 6.6±3.9 | 0.675 |
| **Q4 (9.0-58.2)** | 72.3±10.5 | 76.2±11.1 | 0.250 | 7.5±5.9 | 7.6±4.1 | 0.946 |
|  |  |  |  |  |  |  |
| **TT (nmol/L))** | **Basal** | **Follow up** |  | **Basal** | **Follow up** |  |
| **Q1 (0.3-12.4)** | 69.3±14.6 | 75.6±16.1 | 0.083 | 6.0±3.3 | 7.2±4.0 | 0.399 |
| **Q2 (12.5-15.0)** | 73.3±11.9 | 75.5±11.8 | 0.533 | 6.9±5.7 | 8.2±4.5 | 0.374 |
| **Q3 (15.2-18.1)** | 75.3±9.8 | 78.0±8.3 | 0.449 | 6.9±5.2 | 6.6±3.8 | 0.813 |
| **Q4 (18.3-31.5)** | 77.0±9.2 | 80.2±8.3 | 0.373 | 8.7±5.3 | 8.5±3.9 | 0.893 |
|  |  |  |  |  |  |  |
| **Total Sperm Count (10^6^cells/ejaculate))** | **Basal** | **Follow up** |  | **Basal** | **Follow up** |  |
| **Q1 (0.01-6.51)** | 70.3±14.1 | 73.4±14.3 | 0.387 | 8.9±7.0 | 6.9±3.9 | 0.138 |
| **Q2 (7.65-20.25)** | 72.8±12.2 | 76.4±11.8 | 0.304 | 5.8±3.4 | 7.5±3.5 | 0.199 |
| **Q3 (21.00-32.50)** | 76.1±9.9 | 80.8±9.5 | 0.160 | 7.1±4.4 | 7.9±4.3 | 0.548 |
| **Q4 (33.00-39.60)** | 75.7±10.0 | 78.2±9.2 | 0.508 | 6.7±4.3 | 8.2±4.9 | 0.325 |
|  |  |  |  |  |  |  |
| **Type A Motility (%)** | **Basal** | **Follow up** |  | **Basal** | **Follow up** |  |
| **Q1 (0.0-18.0)** | 66.5±14.1 | 66.7±12.7 | 0.965 | 5.8±5.6 | 5.5±3.5 | 0.813 |
| **Q2 (20.0-33.0)** | 72.5±11.4 | 77.9±8.2 | 0.094 | 6.2±3.9 | 6.9±3.5 | 0.588 |
| **Q3 (35.0-50.0)** | **76.8±9.5** | **82.6±7.7** | **0.046** | 7.6±3.8 | 10.0±4.0 | 0.053 |
| **Q4 (53.0-81.0)** | 80.3±5.2 | 83.1±7.9 | 0.402 | 9.3±6.2 | 7.9±4.0 | 0.322 |
|  |  |  |  |  |  |  |
| **Type A+B Motility (%)** | **Basal** | **Follow up** |  | **Basal** | **Follow up** |  |
| **Q1 (6.0-26.0)** | 65.6 ±14.5 | 67.5±13.1 | 0.542 | 6.2±5.6 | 6.0±3.9 | 0.892 |
| **Q2 (27.0-39.0)** | 74.8±8.9 | 76.4±8.9 | 0.605 | 5.9±3.7 | 7.3±4.2 | 0.300 |
| **Q3 (40.0-55.0)** | **73.9±10.6** | **81.7±8.2** | **0.012** | 6.9±4.1 | 8.8±3.9 | 0.167 |
| **Q4 (57.0-85.0)** | 81.3±5.7 | 84.2±7.4 | 0.366 | 9.7±5.7 | 8.4±3.9 | 0.357 |
|  |  |  |  |  |  |  |
| **Viability (%)** | **Basal** | **Follow up** |  | **Basal** | **Follow up** |  |
| **Q1 (37.0-68.0)** | **58.0±8.3** | **66.6±13.8** | **<0.001** | 5.5±5.3 | 6.7±3.9 | 0.397 |
| **Q2 (69.0-76.0)** | **72.8±2.4** | **77.3±8.9** | **0.042** | 6.8±4.7 | 6.7±3.2 | 0.973 |
| **Q3 (77.0-83.0)** | 80.0±1.7 | 82.0±6.7 | 0.391 | 8.3±4.9 | 9.1±4.2 | 0.552 |
| **Q4 (84.0-91.0)** | 86.6±2.2 | 84.9±3.6 | 0.466 | 8.3±4.9 | 8.2±4.7 | 0.972 |
|  |  |  |  |  |  |  |
| **Typical Morphology (%)** | **Basal** | **Follow up** |  | **Basal** | **Follow up** |  |
| **Q1 (0.0-4.0)** | 69.3±12.2 | 73.1±12.8 | 0.162 | **2.9±1.2** | **5.3±3.4** | **0.001** |
| **Q2 (5.0-6.0)** | 75.1±9.3 | 80.8±8.7 | 0.185 | 5.6±0.6 | 7.4±3.4 | 0.120 |
| **Q3 (7.0-10.0)** | 78.0±12.4 | 79.5±9.7 | 0.692 | 8.4±0.9 | 9.9±3.1 | 0.141 |
| **Q4 (11.0-26.0)** | 77.1±9.7 | 80.5±10.6 | 0.342 | **14.7±3.9** | **9.9±3.6** | **<0.001** |

Abbreviations: Quartile, Q; left, L; right, R; follicle stimulating hormone, FSH; total testosterone, TT.

Significance: significant P values from the follow up to basal comparisons are in bold.

**B** Baseline to follow up variations in sperm viability and morphology, according to distribution quartiles of basal ultrasound parameters in 85 patients undergone to varicocele treatment.

| **Basal parameter** | **Sperm Viability (%)** | | **P value** | **Sperm Morphology (%)** | | **P value** | |
| --- | --- | --- | --- | --- | --- | --- | --- |
| **TTP-R (s)** | **Basal** | **Follow up** |  | **Basal** | **Follow up** |  | |
| **Q1 (11.0-17.1)** | 76.1±11.8 | 77.0±13.0 | 0.793 | 6.4±3.7 | 7.4±3.6 | 0.479 | |
| **Q2 (17.2-19.2)** | 75.5±11.6 | 76.8±11.6 | 0.719 | 7.5±5.2 | 6.8±4.1 | 0.598 | |
| **Q3 (19.3-20.4)** | 71.2±12.3 | 76.3±11.9 | 0.282 | 9.3±7.3 | 8.8±5.4 | 0.789 | |
| **Q4 (20.5-48.3)** | **71.8±12.3** | **78.2±10.7** | **0.035** | 6.5±4.4 | 7.9±3.9 | 0.237 | |
|  |  |  |  |  |  |  |  |
| **MTT-R (s)** | **Basal** | **Follow up** |  | **Basal** | **Follow up** |  |  |
| **Q1 (24.9-31.0)** | 74.4±13.9 | 75.1±13.2 | 0.832 | 6.2±3.5 | 6.4±4.0 | 0.893 |  |
| **Q2 (31.2-34.3)** | 73.1±12.5 | 75.7±10.7 | 0.470 | 6.6±4.1 | 7.5±4.0 | 0.511 |  |
| **Q3 (34.4-38.4)** | 72.0±11.4 | 76.7±13.2 | 0.184 | 7.7±6.0 | 7.7±3.9 | 0.974 |  |
| **Q4 (38.6-66.6)** | 75.6±9.0 | 81.9±7.3 | 0.084 | 8.1±6.1 | 8.9±4.3 | 0.581 |  |
|  |  |  |  |  |  |  |  |
| **Wash in-R (s)** | **Basal** | **Follow up** |  | **Basal** | **Follow up** |  |  |
| **Q1 (5.7-9.1)** | 75.4±10.9 | 75.9±11.2 | 0.867 | 7.5±6.4 | 8.0±4.4 | 0.670 |  |
| **Q2 (9.2-11.2)** | 70.1±13.3 | 76.0±13.8 | 0.102 | 5.8±3.2 | 6.7±3.9 | 0.503 |  |
| **Q3 (11.3-13.2)** | 73.7±13.1 | 76.7±11.2 | 0.405 | 7.9±4.9 | 7.7±4.3 | 0.867 |  |
| **Q4 (13.3-23.3)** | 73.7±11.7 | 80.6±9.8 | 0.174 | 7.3±4.9 | 7.9±3.7 | 0.663 |  |
|  |  |  |  |  |  |  |  |
| **Wash out-R (s)** | **Basal** | **Follow up** |  | **Basal** | **Follow up** |  |  |
| **Q1 (9.9-22.0)** | 73.1±11.6 | 77.2±11.3 | 0.242 | 6.9±4.1 | 7.4±3.3 | 0.742 |  |
| **Q2 (22.2-25.4)** | 74.3±10.8 | 80.5±9.9 | 0.084 | 6.9±4.2 | 7.8±4.9 | 0.545 |  |
| **Q3 (25.5-28.8)** | 70.9±15.1 | 72.4±13.7 | 0.679 | 6.7±4.3 | 6.3±3.0 | 0.788 |  |
| **Q4 (28.9-55.0)** | 76.5±8.6 | 79.0±9.9 | 0.488 | 8.0±7.0 | 9.0±4.6 | 0.480 |  |
|  |  |  |  |  |  |  |  |
| **TTP-L (s)** | **Basal** | **Follow up** |  | **Basal** | **Follow up** |  |  |
| **Q1 (14.5-18.5)** | 77.9±11.3 | 78.6±11.1 | 0.849 | 6.9±3.4 | 7.6±3.7 | 0.640 |  |
| **Q2 (18.6-20.6)** | 70.3±11.4 | 71.3±12.9 | 0.781 | 7.9±6.3 | 7.4±3.9 | 0.969 |  |
| **Q3 (20.9-22.6)** | 74.9±12.5 | 79.8±10.8 | 0.160 | 7.4±5.2 | 8.3±4.9 | 0.505 |  |
| **Q4 (22.8-35.2)** | **71.9±11.0** | **79.8±9.6** | **0.027** | 6.2±4.5 | 7.1±3.9 | 0.526 |  |
|  |  |  |  |  |  |  |  |
| **MTT-L (s)** | **Basal** | **Follow up** |  | **Basal** | **Follow up** |  |  |
| **Q1 (25.5-33.6)** | 71.8±14.1 | 73.7±13.8 | 0.606 | 6.7±3.9 | 6.5±3.5 | 0.733 |  |
| **Q2 (34.0-41.1)** | 76.4±10.5 | 77.5±12.6 | 0.747 | 7.5±5.8 | 7.0±4.9 | 0.527 |  |
| **Q3 (41.5-44.5)** | **71.7±11.5** | **79.2±9.3** | **0.033** | 6.6±4.4 | 7.3±4.4 | 0.335 |  |
| **Q4 (45.4-60.4)** | 75.1±10.6 | 78.8±9.7 | 0.323 | 7.8±5.6 | 8.8±5.1 | 0.154 |  |
|  |  |  |  |  |  |  |  |
| **Wash in-L (s)** | **Basal** | **Follow up** |  | **Basal** | **Follow up** |  |  |
| **Q1 (7.1-9.1)** | 73.8±10.2 | 76.1±13.1 | 0.618 | 7.0±6.5 | 7.6±4.8 | 0.735 |  |
| **Q2 (9.3-12.3)** | 72.6±14.2 | 76.7±12.9 | 0.183 | 7.2±4.3 | 7.8±3.9 | 0.636 |  |
| **Q3 (12.5-15.4)** | 73.3±10.6 | 77.1±10.9 | 0.302 | 6.9±4.0 | 6.8±3.4 | 0.947 |  |
| **Q4 (15.6-34.3)** | 75.7±10.2 | 79.1±9.6 | 0.347 | 7.3±6.1 | 8.1±4.6 | 0.550 |  |
|  |  |  |  |  |  |  |  |
| **Wash out-L (s)** | **Basal** | **Follow up** |  | **Basal** | **Follow up** |  |  |
| **Q1 (15.8-24.3)** | 76.1±8.2 | 78.8±9.3 | 0.456 | 7.1±4.3 | 7.4±4.7 | 0.816 |  |
| **Q2 (24.4-28.9)** | 70.9±17.2 | 72.3±14.3 | 0.696 | 7.5±5.4 | 6.8±3.0 | 0.603 |  |
| **Q3 (29.2-33.4)** | 73.6±9.3 | 78.8±9.9 | 0.141 | 6.8±5.9 | 7.8±4.2 | 0.464 |  |
| **Q4 (33.5-47.6)** | 74.5±9.9 | 79.8±11.1 | 0.158 | 7.1±4.4 | 8.5±4.4 | 0.345 |  |

Abbreviations: Quartile. Q; left. L; right. R; time to peak. TTP; mean transit time, MTT.

Significance: significant P values from the follow up to basal comparisons are in bold.
