# Supplementary material for: Predictive parameters of semen outcome improvement after varicocele treatment by scleroembolization: a retrospective study
Source: J Endocrinol Invest. 2026 Apr 29;49(7):1565–80. doi: 10.1007/s40618-026-02844-0 (PMC13451323; doi:10.1007/s40618-026-02844-0)
Supplement: Supplementary file 2 [file 40618_2026_2844_MOESM2_ESM.docx]

**Supplemental Table S2**

| **A** | **Output variable: Total Sperm Count Doubling** | | |
| --- | --- | --- | --- |
| **Parameter** | **P Value** | **Odd Ratio** | **95% C.I.** |
| Procedure | 0.865 | 0.922 | 0.362-2.351 |
| Grade-L II | 0.510 |  |  |
| Grade-L III | 0.943 | 1.075 | 0.152-7.622 |
| Grade-L IV | 0.832 | 1.233 | 0.177-8.572 |
| Grade-L V | 0.284 | 3.510 | 0.352-34.998 |
| Grade-R 0 | 0.878 |  |  |
| Grade-R I | 0.616 | 1.504 | 0.305-7.419 |
| Grade-R II | 0.903 | 1.197 | 0.066-21.815 |

| **B** | **Output variable: Total Motile Sperm Count Doubling** | | |
| --- | --- | --- | --- |
| **Parameter** | **P Value** | **Odd Ratio** | **95% C.I.** |
| Procedure | 0.810 | 0.892 | 0.352-2.262 |
| Grade-L II | 0.571 |  |  |
| Grade-L III | 0.568 | 0.565 | 0.080-3.997 |
| Grade-L IV | 0.627 | 0.618 | 0.089-4.290 |
| Grade-L V | 0.688 | 1.602 | 0.161-15.968 |
| Grade-R 0 | 0.938 |  |  |
| Grade-R I | 0.721 | 1.337 | 0.272-6.558 |
| Grade-R II | 0.999 | 17🞨10^8^ | 0.000-N/D |

Logistic regression analysis of basal left (L) and right (R) varicocele grading, according to Penile Imaging Working Group of the European Society of Urogenital Radiology, predicting total sperm count or total motile sperm doubling at follow-up.

Abbreviations: left, L; right, R;
